# Supplementary material for: The challenge of SARS-CoV-2 environmental monitoring in schools using floors and portable HEPA filtration units: Fresh or relic RNA?
Source: PLoS One. 2022 Apr 22;17(4):e0267212. doi: 10.1371/journal.pone.0267212 (PMC9032406; doi:10.1371/journal.pone.0267212)
Supplement: S1 File — S1 Table shows the RT-qPCR standard curve and S2 Table lists the samples used in validation. (DOCX) [file pone.0267212.s001.docx]

**Supplementary information**

**The challenge of SARS-CoV-2 environmental monitoring in schools using floors and portable HEPA filtration units: Fresh or relic RNA?**

Rogelio Zuniga-Montanez^1^, David A. Coil^2^, Jonathan A. Eisen^2, 3, 4^, Randi Pechacek^1^, Roque G. Guerrero^1^, Minji Kim^1^, Karen Shapiro^5^, Heather N. Bischel^1^

^1^Department of Civil and Environmental Engineering, One Shields Avenue, University of California, Davis, CA, 95616, USA

^2^Genome Center, University of California, Davis

^3^Department of Medical Microbiology and Immunology, School of Medicine, University of California, Davis

^4^Department of Evolution and Ecology, University of California, Davis

^5^Department of Pathology, Microbiology and Immunology, School of Veterinary Medicine, University of California, Davis

**Running title:** SARS-CoV-2 environmental monitoring

***Corresponding author:**

Heather Bischel

[hbischel@ucdavis.edu](mailto:hbischel@ucdavis.edu)

# S1 Supplementary Methods

## S1.1 Validation sampling for the environmental detection of SARS-CoV-2 on surfaces and HEPA filtration units

We validated the detection of SARS-CoV-2 through swabbing surfaces and HEPA air filter units in two locations in Northern California after known exposures . The first location was a house where a visitor stayed for a period of 2 hours, mostly wearing a mask with the exception of ~20 minutes to have a meal. The visitor received a positive COVID-19 test result one day after the visit. We obtained swab samples from different surfaces in the dining, living and bathrooms four days after the exposure (Table S2). At the second location, which was a school classroom, two students tested positive for COVID-19 before the cohort was quarantined. We sampled surfaces two and six days after the students were on campus (Table S2). A portable air purifier equipped with an H13 HEPA filter (MA-40, Medify Air, USA) that was active in the room when the positive individuals were present was dismantled and parts of it were sampled three days after. Three samples each were collected from the outer unit grill cover and the pre-filter mesh, and four samples were collected from the H13 HEPA filter surface (Table S2).

## S1.2 SARS-CoV-2 floor and HEPA filter sampling instructional videos

Floor sampling instructional video can be found here: <https://youtu.be/HuOuzR9Rpg8>

HEPA filter sampling instructional video can be found here: <https://youtu.be/MzV8tDMKsZc>

## S1.3 Detailed RNA extraction protocols

We used two methods to extract the SARS-CoV-2 RNA from surface and HEPA filter samples, one manual and one automated. Section 2.4 details the periods of time when each method was utilized. For the manual extractions with the PureLink Viral RNA/DNA kit (Thermo Fisher Scientific, USA), sample volumes of 200 μl were mixed with 25 μl of Proteinase K and 200 μl of Lysis Buffer. The resulting solutions were vortexed for 15 seconds and incubated at 56 °C for 15 minutes. 250 μl of 100% ethanol was added, vortexed for 15 seconds and incubated at room temperature for 5 minutes. The solutions were centrifuged for 20 seconds at 10,000 x g to remove foam. The lysates (~675 μl) were transferred to Viral Spin Columns in collection tubes and centrifuged for 1 minute at 6,800 x g. The Spin Columns were placed in new Wash Tubes. 500 μl of Wash Buffer (WII with ethanol) was added to the Spin Columns and centrifuged for 1 minute at 6,800 x g. The flow through was discarded, and the 500 μl Wash Buffer (WII with ethanol) wash and centrifugation were repeated. The Spin Columns were placed in clean Wash Tubes and centrifuged for 1 minute at maximum speed (~20,000 x g) to remove residual Wash Buffer. The Spin Columns were placed in sterile microcentrifuge tubes and 50 μl of sterile RNase-free water was used to elute the RNA extracts. The samples were incubated at room temperature for 1 minute and centrifuged at maximum speed to elute the RNA. The extracts were stored in a -80 °C freezer.

The MagMAX Microbiome Ultra Nucleic Acid Isolation Kit (Applied Biosystems, USA) and the KingFisher Flex purification system (Thermo Fisher Scientific, USA) were used for automated RNA extractions. Sample volumes of 200 μl were transferred to 96-Deep Well Kingfisher plates. A solution that contained 250 μl of MagMAX Viral/Pathogen Binding Solution and 10 μl of MagMAX DNA/RNA Binding Beads per sample was made and 260 μl of the solution was added to each well of the 96-Deep Well plates containing samples. Wash 1 and Wash 2 Kingfisher plates were prepared with 1 ml of MagMAX Viral/Pathogen Wash Solution per well. Wash 3 and Wash 4 plates were prepared with 1 ml of 80% ethanol per well. Elution plates were prepared with 100 μl of MagMAX Viral/Pathogen Elution Buffer in each well. The plates were loaded into the Kingfisher and the MagMAX_Microbiome_Stool_Flex.bdz extraction protocol was run. The extracted samples were transferred to microcentrifuge tubes for storage at -80 °C.

## S1.4 RT-qPCR standard curves

Standard curves were created for the spike glycoprotein (S) and φ6 bacteriophage assays. Known copy numbers of SARS-CoV-2 RNA obtained from clarified viral supernatants of heat inactivated virus donated by the University of Oregon were used for the SARS-CoV-2 RNA standard curve. Four replicates of ten-fold serial dilutions of viral RNA in the range of 3.2x10^5^ to 3.2 copies per μl were used for generating the standard curve (Table S1). φ6 bacteriophage was prepared as described by Safford et al. (2022). Positive extraction controls were prepared weekly by spiking 9 ul of the φ6 stock into a sample tube that contained 1.5 ml of DNA/RNA shield and the swab collected from an air filter in the laboratory. The control sample was vortexed, and 200 ul was transferred to each kingfisher plate for extraction alongside samples. Extracts containing φ6 were stored at -80°C prior to analysis in batch. The primers and probes for each assay are detailed in Table 1 and the thermal cycling conditions described in section 2.4.

The lower limit of quantification was determined as the lowest concentration that yielded precise amplification in standard curve regression analysis. We considered samples to be detected but not quantifiable if Ct<40, and not detected if Ct>40. Replication of the standard curve and lower limit of quantification after termination of the analysis indicated stability of the standard curves through time.

# S2 Supplementary tables

Table S1. RT-qPCR standard curve for SARS-CoV-2 using viral RNA.

| **Target** | **Standard curve** | **R^2^** | **Efficiency^a^** | **Limit of quantification** |
| --- | --- | --- | --- | --- |
| SARS-CoV-2  S gene | y= -3.218x + 39.052 | 0.999 | 104.53% | 3.2 copies/μl of extract |
| φ6 bacteriophage | y=-2.961x + 38.822 | 0.998 | 117.65% | 11.6 copies/μl of extract |

^a^PCR efficiency = 10^-1/slope^-1

Table S2. Samples tested during the validation experiments for the detection of SARS-CoV-2 on surfaces and HEPA air purification units after known exposures.

| **Location** | **Sampling conducted x days after exposure** | **Surface sampled** | **Number of samples collected** | **SARS-CoV-2 positive samples** |
| --- | --- | --- | --- | --- |
| House | 4 | Underside of chair | 1 | 1 |
|  |  | Floor under chair | 1 | 1 |
|  |  | Window sill | 1 | 0 |
|  |  | Wall | 1 | 0 |
|  |  | Bathroom counter | 1 | 0 |
|  |  | Video game controller | 1 | 0 |
|  |  | Front door | 1 | 0 |
|  |  | Dining table | 1 | 0 |
|  |  | HVAC filter | 2 | 0 |
| School | 2 | Underside of positive student's desk | 1 | 1 |
|  |  | Underside of positive student's chair | 1 | 1 |
|  |  | Underside of positive student's tool box | 1 | 1 |
|  |  | Fire extinguisher box | 1 | 0 |
|  |  | Bookcase shelf | 1 | 0 |
|  |  | Projector | 1 | 0 |
|  | 3 | Plastic grill covering HEPA filter unit | 3 | 1 |
|  |  | Pre-filter mesh | 3 | 3 |
|  |  | H13 HEPA filter surface | 4 | 2 |
|  | 6 | Underside of positive student's desk | 1 | 1 |
|  |  | Underside of positive student's chair | 1 | 1 |
|  |  | Underside of positive student's tool box | 1 | 1 |
|  |  | 2' floor perimeter of positive student's desk | 4 | 0 |
|  |  | 4' floor perimeter of positive student's desk | 4 | 0 |
|  |  | Floor (middle of classroom) | 1 | 0 |
|  |  | Door push bar | 1 | 0 |
|  |  | 2 light switches and backplate | 1 | 0 |
|  |  | Hand sanitizer | 1 | 0 |
|  |  | Faucet handle | 1 | 0 |
|  |  | Desk near positive student's desk | 1 | 0 |
|  |  | Whiteboard magtray | 1 | 0 |
|  |  | Chair behind positive student | 1 | 0 |
|  |  | Chair in front of positive student | 1 | 0 |
