# Supplementary material for: The challenge of SARS-CoV-2 environmental monitoring in schools using floors and portable HEPA filtration units: Fresh or relic RNA?
Source: PLoS One. 2022 Apr 22;17(4):e0267212. doi: 10.1371/journal.pone.0267212 (PMC9032406; doi:10.1371/journal.pone.0267212)
Supplement: Attachment — Submitted filename: airfilterpaper_response_to_review_v1.docx [file pone.0267212.s002.docx]

**Response to Reviewers**

We are submitting here the revised version of our manuscript “The challenge of SARS-CoV-2 environmental monitoring in schools using floors and portable HEPA filtration units: Fresh or relic RNA?”. We thank the editor and reviewers for their detailed comments and suggestions and have made revisions in response to these.

*Journal Requirements:*

*When submitting your revision, we need you to address these additional requirements.*

*1. Please ensure that your manuscript meets PLOS ONE's style requirements, including those for file naming. The PLOS ONE style templates can be found at*

*https://journals.plos.org/plosone/s/file?id=wjVg/PLOSOne_formatting_sample_main_body.pdf and*

[*https://journals.plos.org/plosone/s/file?id=ba62/PLOSOne_formatting_sample_title_authors_affiliations.pdf*](https://journals.plos.org/plosone/s/file?id=ba62/PLOSOne_formatting_sample_title_authors_affiliations.pdf)

We have double-checked that our manuscript meets the PLOS ONE style requirements

*2. Please amend your current ethics statement to address the following concerns:*

*a) Did participants provide their written or verbal informed consent to participate in this study?*

*b) If consent was verbal, please explain i) why written consent was not obtained, ii) how you documented participant consent, and iii) whether the ethics committees/IRB approved this consent procedure."*

We have updated the ethics statement as requested.

*3. Thank you for stating the following in the Acknowledgments Section of your manuscript:*

*“Funding was provided by Healthy Yolo Together/Healthy Davis Together, a partnership between the University of California, Davis, the city of Davis, and Yolo county.”*

*We note that you have provided additional information within the Acknowledgements Section that is not currently declared in your Funding Statement. Please note that funding information should not appear in the Acknowledgments section or other areas of your manuscript. We will only publish funding information present in the Funding Statement section of the online submission form.*

*Please remove any funding-related text from the manuscript and let us know how you would like to update your Funding Statement. Currently, your Funding Statement reads as follows:*

*“Funding was provided by Healthy Yolo Together/Healthy Davis Together. The funders had no role in study design, data collection and analysis, decision to publish, or preparation of the manuscript.”*

We have removed the funding information from the Acknowledgements section of the paper and have updated the funding statement accordingly.

*Please include your amended statements within your cover letter; we will change the online submission form on your behalf.*

*4. Please include captions for your Supporting Information files at the end of your manuscript, and update any in-text citations to match accordingly. Please see our Supporting Information guidelines for more information:* [*http://journals.plos.org/plosone/s/supporting-information*](http://journals.plos.org/plosone/s/supporting-information)*.*

This has been corrected.

Specific responses below:

*Reviewer #1: This is a very interesting manuscript, but it is necessary to focus on a few problematic passages.*

*The methodology, not the supplementary materials, should indicate which portable air purifier was used. For used air purifier should be verified the air flow in cubic meters per hour and should be indicated how long the air has been sampled. The authors should calculate the approximate area of the sampled part of the HEPA filter in square centimeters.*

While we appreciate the value of such data, this was not a controlled experiment. The teachers in the classrooms had control (undocumented) over when the units were used. They were supposed to run the air purifiers any time people were in the room but there is of course variation. The portable air purifier models used in each school are indicated in the Methods and in Table 1. The manufacturers report clean air delivery rates (CADR) of 380 m3/h for the Medify MA-40, and ratings of 319-333 m3/h for the AeraMax® 300. This information was added in the Methods section. The area of the most common filter units that was sampled is about 823 cm^2^ and we have added this information to the Methods.

*Why did the authors used the Spike protein RNA detection method? It is the most mutating part of SARS-CoV-2 coronavirus and the accuracy and sensitivity of some methods using spike protein detection is low. Can the authors analyze the primers and probes using the GISAID database and determine if the primers and probes are designed for regions that are not mutated? This information should be provided in Methods.*

We agree that the spike glycoprotein region is highly variable. At the time when we started establishing a protocol for environmental sampling in the Fall of 2020, colleagues of ours had success at detecting the virus on heating, ventilation, and air conditioning (HVAC) systems using the primer set used in our study (Horve et al., 2021). We decided to follow a similar protocol as it was of great importance we provided prompt testing resources to our community. While our primers can (*in silico*) have reduced binding to some variants, none of those variants were relevant to the time period under investigation.

*Could the authors create a calibration line for RealTime PCR according to the standard and realistically calculate the number of detected viruses (RNAs) in each PCR positive reaction? A table with the numbers of viruses in each sample should be provided, not Ct values. It is not possible to compare Ct values between different methodologies. The introductory paragraph of Chapter 3.1 is a bit confusing, as each methodology has a different Ct value for capturing the same amount of DNA. Therefore, Ct values cannot be compared. This paragraph should be redrafted in this respect.*

We appreciate this comment and had similar discussions ourselves when drafting the paper. We decided to report Ct values for two main reasons; comparisons to other work, and the fact that we didn’t rigorously control for the sampled surface area at collection.

At the time of this work, most groups were reporting their data in terms of Ct values (Marshall et al. 2020) which makes the results as presented here most directly comparable. Discussions with two different commercial providers of this kind of service confirmed that they were using Ct “action thresholds” when swabbing in schools and workplaces. In addition, because the surface area for swabbing was not rigorously controlled, a calculation of the number of detected viruses would not be more informative than the Ct values presented here. In fact, it could give a misleading impression of accuracy for this reason. We provided a RT-qPCR standard curve in the Supplemental Material (Table S1) determined from replicate serial dilutions of SARS-CoV-2 viral RNA (Figure S4) to facilitate calculation of genome copies per reaction should other researchers seek these units. The main messages here (that the virus was present at only low levels and that relic RNA is a problem) would not change with a conversion to the number of detected virions.

*If the analysis of some rooms showed positivity alternating with negativity, how did the authors rule out PCR contamination in the laboratory or at collection? It should be described in manuscript.*

Because these samples were collected by personnel at the schools, we had little control over potential contamination at that step, beyond wearing masks (which were required at the time) and reminders to wear gloves . However, the pattern of data positivity seen in our study does not suggest contamination on-site by personnel collecting the specimens.

Regarding lab contamination, we used nuclease-free water as a negative control for RT-qPCR, as well as running an extraction blank in most runs of the Kingfisher. More details regarding negative controls have been added to the Methods section. All laboratory reagent blank controls tested negative by RT-qPCR, supporting lack of contamination in our testing workflow.

*Is it really a reusable air purifier that captured coronavirus RNA even after changing the filter? How did authors decontaminate from the RNA mesh and cover where RNAs can also be captured?*

The entire filter with mesh and housing was replaced after each positive detection.

*How is the cleaning done in the classrooms – every day? By which method? Is the disinfectant used for floor cleaning and if so, which one? It is possible that the same mop is used in toilets and this mop can then spread viral RNA in other rooms in the school. For example, one of the school staff could be positive and spread viruses in the toilets.*

This information varied widely across schools and even classrooms within a school, and was also not documented by school staff. Yes, it is possible that viral RNA could be transmitted between locations in the manner described, but this would not explain the patterns of classrooms that tested positive in this study. The potential impact of “outsider” RNA on the results obtained is discussed in the manuscript.

*The whole section 3. Results and discussion I recommend to rewrite to two chapters: the results chapter of the discussion chapter. Unfortunately, this chapter turned out to be confusing for readers. The reader will not know whether these are the results of this work or a citation of other results. The separate chapter Results will greatly contribute to the clarity of the results.*

After careful consideration of this suggestion, our team has elected to keep the formatting of the results and discussion as a cohesive section to more clearly link interpretation of our results to each part of the study. We made modifications throughout section 3 to provide clarity between results from this study and referenced data and reduce potential confusion for the reader. Our hope is that the “Conclusions” section ties everything together.

*The abstract cannot state: "The high-Ct positive results from environmental swabs further suggest the absence of active infections", if the authors did not determine the viability of the virus. The abstract should indicate the number of viruses, not the Ct values.*

There is an established understanding that environmental samples with high Ct values do not tend to contain infectious virus, specifically samples with Ct values above 30 (Zhou et al. 2020; Krambrich et al. 2021). We discussed this point in the manuscript and we feel this statement is merited. We made a modification of the sentence to indicate that high-Ct environmental detections made in the absence of known active infections supports our conclusions.

*If the abstract compares air sampling and square centimeters on the floor, for the air sampling should indicate the air flow (cubic meters per hour) through the HEPA filter and samples surface of the HEPA filter.*

As stated above, it was not possible to calculate this information across dozens of classrooms with different HVAC systems and no control over the air purification units usage. We added CADRs reported by the manufacturers for the HEPA filters where we were able to obtain this information.

*The methodology lacks an indication how children and teachers were tested for positivity.*

The clinical testing (saliva-based RT qPCR) was not a part of this study, it was offered through a community program that is referenced at the start of Section 2.5.

*Missing is used RealTime PCR method and the sampling method. Saliva, oropharyngeal swabs, nasopharyngeal swabs?*

See the references at the start of Section 2.5 which contain links to information detailing the clinical testing.

*Reviewer #2: This work is an important research methodology that shows the possibility of SARS-CoV-2 surface detection (surveillance) from floors and portable filters. I enjoyed reading the manuscript, and I have a few minor comments below that I believe will help improve the manuscript. I accept this manuscript for publication after addressing my comments below.*

*Introduction*

*1. First paragraph. Introduce COVID-19 in one sentence.*

Done

*2. First paragraph, last sentence, add a reference.*

This statement is intended as a rationale for doing this work, not as an established fact. The following paragraph contains numerous references to the use of surface sampling during the pandemic.

*3. Paragraph 3: Wastewater surveillance cannot provide information about virus presence in interior spaces (e.g., location inside the building floors and rooms). That is correct. However, you did not mention the latest study by Sousan et al. 2021 (below) that suggests sampling HVAC systems as a surveillance method. You mention the HVAC system in the next paragraph. How does this work address the limitation(s) or difference(s) in Sousan et al. (2021)?*

*Sousan, S., Fan, M., Outlaw, K., Williams, S., & Roper, R. L. (2021). SARS-CoV-2 Detection in Air Samples from Inside Heating, Ventilation, and Air Conditioning (HVAC) Systems-COVID Surveillance in Student Dorms. American journal of infection control.*

This article had not yet come out when we were writing this section of the paper and is indeed worth discussing. We have added references to this study in both the Introduction and Results/Discussion. There is a discussion of the limitations of this approach in the last paragraph of Section 3.1.

*Materials and Methods*

*4. Section 2.2: I suggest removing the words “Pilot study.” Five schools from January to August is a lot of work.*

We have removed all 5 instances of the word “pilot” throughout the manuscript

*Results*

*5. Before you start the sections, it would be nice to mention the total number of samples collected and maybe a breakdown of each method and location by sample. Before you mention the details, this will show the magnitude of your work*.

We have added the total number of samples to the Abstract, Introduction, and Conclusion as well as the number of samples from each school in the section describing that school.

*6. Section 3.1 (first paragraph): The Methods section should mention the ten surface swab samples.*

These swabs are included in the 46 swabs described in the third sentence of section 2.1 in the Methods.

*7. Section 3.1 (first paragraph): I am confused about this sentence “First, we sampled surfaces at a house where an asymptomatic person who later tested positive for COVID-19 was present for 2 hours.” In the previous sentence, you mention “at least one COVID-19 positive individual was known”. Why did you sample in that house if you did not know the person had COVID-19 (asymptomatic)? Sampling in a school makes sense because of the students’ exposure between their home and the classroom. Therefore, the likelihood of detecting COVID-19 is high. Was sampling in this house randomly, and how did you choose this random house? Also, in the Methods section, you mention “using opportunistic sampling in two locations within six days after one or more clinical COVID-19 cases were identified”. So in the Methods, you mention that sampling started after identifying COVID-19, and here you say that you sampled in the house and then later you found out that the person had covid-19. Maybe there is a wording issue; please re-write for clarity. Please also address this comment in the Methods section.*

This was confusingly worded and has been clarified in the manuscript text. In both the house and the school, sampling occurred after we were notified about a positive case in those locations (both asymptomatic).

*8. Section 3.1 (second paragraph): Did you swab underneath the chairs for all students randomly or just the ones that tested positive?*

We swabbed only the chairs that we knew belonged to positive cases since at this point we were not conducting surveillance, just trying to validate the method.

*9. Section 3.1 (third paragraph): give an example using the Sousan et al. (2021) study (above) to validate your point.*

The Sousan approach is different from work we had previously cited in that Sousan et al. placed the air samplers into the HVAC system and not the living space. We have now addressed this in a few places in this section.

*10. Section 3.2 (first paragraph: last four sentences): Is this a false-positive result, and is this considered a limitation of the surveillance method? Please clarify.*

This depends on how one defines “false positive”. It is our belief that most people reserve this term for technical false positives (e.g. contamination of PCR reagents) which we do not believe occurred. It’s a “false positive” only in the sense that the detection of RNA doesn’t necessarily correlate with active recent shedding (relic RNA), but that’s a limitation of the entire approach and is discussed at length in the paper. We have added a sentence to the end of this paragraph to clarify.

*11. Figures 1 and 2 are blurry and sufficient for publication. Please improve the quality of the figure considerably and resubmit to the journal.*

We have included high-resolution figures for the resubmission

*12. In the end, how does this work compare to other SARS-CoV-2 surveillance methods? Please discuss?*

One of the challenges with the sampling period for this study was the very low positivity rate for COVID-19 in the surveilled schools. One of our original objectives was to evaluate this method against others, but given the low positivity rates, we were unable to draw conclusions regarding the relative utility of this method vs others. We discuss relic and outsider RNA at length, as this appeared to be a primary explanation for the positive environmental samples we detected. We don’t feel it’s appropriate to speculate on the utility of the method otherwise.

*Conclusion*

*13. Please add the number of positive samples (your PCR results) and the number of confirmed positive samples (clinical PCR testing) for your positives.*

We have added both the total number of swabs collected, as well as the number of classroom sampling events that resulted in a PCR positive to the Conclusion section. Because the clinical test results didn’t correlate with our environmental detection (and we also don’t have access to all clinical data from the schools) we did not include this information here.
